# Segregation of Maghemite Nanoparticles within Symmetric Diblock Copolymer and Triblock Terpolymer Patterns under Solvent Vapor Annealing

**DOI:** 10.3390/ma13061286

**Published:** 2020-03-12

**Authors:** George Zapsas, Dimitrios Moschovas, Konstantinos Ntetsikas, Andreas Karydis-Messinis, Nikolaos Chalmpes, Antonios Kouloumpis, Dimitrios Gournis, Nikolaos E. Zafeiropoulos, Apostolos Avgeropoulos

**Affiliations:** Department of Materials Science Engineering, University of Ioannina, University Campus-Dourouti, 45110 Ioannina, Greece; georgios.zapsas@kaust.edu.sa (G.Z.); dmoschov@cc.uoi.gr (D.M.); konstantinos.ntetsikas@kaust.edu.sa (K.N.); karydis.and@gmail.com (A.K.-M.); chalmpesnikos@gmail.com (N.C.); antoniokoul@gmail.com (A.K.); dgourni@uoi.gr (D.G.)

**Keywords:** hybrid materials, block copolymer scaffold, block copolymer, maghemite nanoparticles, solvent vapor annealing, diblock copolymers, triblock terpolymers

## Abstract

Block copolymers (BCPs), through their self-assembly, provide an excellent guiding platform for precise controlled localization of maghemite nanoparticles (MNPs). Diblock copolymers (di/BCP) represent the most applied matrix to host filler components due to their morphological simplicity. A series of nanocomposites based on diblock copolymer or triblock terpolymer matrices and magnetic nanoparticles were prepared to study and compare the influence of an additional block into the BCP matrix. MNPs were grafted with low molecular weight polystyrene (PS) chains in order to be segregated in a specific phase of the matrix to induce selective localization. After the mixing of the BCPs with 10% *w*/*v* PS-*g*-MNPs, nanocomposite thin films were formed by spin coating. Solvent vapor annealing (SVA) enabled the PS-*g*-MNPs selective placement within the PS domains of the BCPs, as revealed by atomic force microscopy (AFM). The recorded images have proven that high amounts of functionalized MNPs can be controllably localized within the same block (PS), despite the architecture of the BCPs (AB vs. ABC). The adopted lamellar structure of the “neat” BCP thin films was maintained for MNPs loading approximately up to 10% w/v, while, for higher content, the BCP adopted lamellar morphology is partially disrupted, or even disappears for both AB and ABC architectures.

## 1. Introduction

Among hybrid nanocomposites, the combination of block copolymers (BCPs) and nanoparticles (NPs) is regarded to be an ideal candidate for the “bottom-up” fabrication of nanocomposites and it remains an area of continuous research. Nanodevice fabrication by BCP/NPs mixtures has been developed due to their delicate size and shape leading to the ability of tailoring the components properties for the corresponding hybrids [1]. The structural control of BCP/NPs makes them suitable for applications, such as magnetic [2], electronic, storage devices [3], and catalysis [4,5,6]. The tuning of the aforementioned properties is directly attributed to the exceptional surface to volume ratio of the dispersed phase as well as the ability of the BCP host to self-assemble into periodic nanodomains. Enthalpic and entropic factors both determine the spatial placement of the nanoparticles to the final BCP/NPs nanocomposites, which is extensively described in the literature [7,8,9,10,11,12,13,14,15,16,17,18]. More specifically, the NPs dispersion and localization on a specific block of the BCP host or on the interface between the different blocks is dependent on balancing the conformational entropy of the BCP host, the inclusion enthalpy of the NPs, as well as the NPs translational entropy [19,20]. Such behavior of the NPs induces morphological changes on the BCP host, which depends on the chemical affinity between the NPs and the chemically different domains of the BCP host. It is well known that the molecular characteristics, composition of the different blocks, and the Flory Huggins interaction parameter (χ) of the adjacent blocks govern the adopted morphology for the neat BCP host [19]. It is evident that the exploitation of the physical/chemical properties of the nanocomposites depends on the precise functionality of the NPs and their controlled incorporation into specific domains of the microphase separated BCPs.

There are many scientific results that are reported in the literature concerning magnetic NPs as embedding fillers into BCP matrices [13,17,20,21,22,23], but they are limited to the MNPs encapsulation within AB- [24,25,26] or ABA-type BCPs [27,28]. Taking advantage of the BCPs periodic structures, higher complexity arises upon the transition from two to three chemically different domains for the BCP host matrix (going from AB diblock copolymers to ABC triblock terpolymers). In the ABC, terpolymer hosts additional factors that are related to volume fraction (φ_A_ + φ_Β_ + φ_C_ = 1, instead of φ_A_ + φ_Β_ = 1, which is the case for the AB copolymers) and to the Flory–Huggins interaction parameters (χ_AB_, χ_AC_, χ_BC_ for the ABC case instead of just χ_AΒ_ for the AB diblock copolymer) determine the size and the type of the final adopted morphology. The phase behavior of the BCPs in bulk [29,30] depends on parameters that are different when the same BCPs are studied as thin films. The type of substrate, film thickness, solvent selectivity, and annealing procedure significantly affect the observed nanostructure [31] on the final thin films of the BCPs. The solvent vapor annealing (SVA) method is the one that is mostly used in all recent studies, since this method improves the kinetics of self-assembly by plasticizing the polymer with the incorporation of the solvent molecules [31].

Referring to the ABC-type MNP/BCPs, Weisner’s group synthesized ABC-iron oxide hybrid materials that consisted of PEP-*b*-PEO-*b*-PHMA {poly[(Ethylene-alt-propylene)-*b*-(Ethylene oxide)-*b*-(*n*-Hexyl methacrylate)]} by selectively incorporating aluminosilicate particles that were confined on the PEO domains, leading to PEO-aluminosilicate phases that formed hexagonally patterned layers that were aligned parallel to the surface of the film [32]. Furthermore, Kortaberria’s group presented the surface placement of PS- and PMMA-grafted maghemite NPs into chemically similar domains by using PS-*b*-PB-*b*-PMMA [polystyrene-*b*-poly(butadiene)-*b*-poly(methyl methacrylate)] as a host BCP matrix [23]. Upon replacing the maghemite NPs with silver NPs within the same BCP matrix, noticeable morphological changes were identified on the final nanocomposite films [33]. These research studies reflect the current interest on nanocomposites that are comprised of higher complexity matrices, such as ABC-type BCPs. Instead, these studies mostly focused on the final induced morphology, while the time-dependent evolution of the film formation has not been excessively studied. Even for low degree of polymerization, the segment immiscibility facilitates the relatively high values of the Flory–Huggins interaction parameter, χ, between adjacent blocks [34].

In most of the aforementioned published studies, the success of NPs encapsulation is determined by their selective decoration into one of the two domains. Beyond the case of di/BCPs, similar NPs sequestration within triblock terpolymers (tri/BCPs) remains limited, even though their wider variety of nanostructures can be potentially useful for microlectronic device applications.

In the present study, we synthesized not only diblock copolymers of the PS-b-PB type (where PB is poly(butadiene)), but also triblock terpolymers consisting of polystyrene and two polydienes: poly(isoprene) or PI and poly(butadiene) or PB. The triblock terpolymers displayed strongly incompatible and microphase separated components, (PS, PB_1,4_, and PI_3,4_), where the two polydienes exhibit specific geometric isomerisms (high −1.4 for PB (~92%) and relatively high −3.4 for PI (~60%)). More specifically, PB exhibits approximately 92% −1.4 microstructure and 8% −1.2, whereas the PI is enriched in −3.4 microstructure (~60%), ~25% corresponds to −1.4, and ~15% to −1.2 microstructures, respectively [35,36,37]. The incorporation of iron oxide NPs (IONPs) and how their presence affects the generated terpolymer/NPs hybrid structure in comparison to corresponding diblock copolymer/NPs systems is discussed. The phase separation in the terpolymers that we synthesized is attributed to the different values of the Flory-Huggins interaction parameters between PS/PB, PS/PI, and PB/PI. The “grafting to” method of PS chains onto the particles’ surface was applied to increase phase selectivity and to avoid maghemite NPs aggregation. Prior to NPs embedding, PS-*b*-PB and PS-*b*-PB_1,4_-*b*-PI_3,4_ BCPs exhibited either two-phase alternating lamellae or three-phase four-layer lamellae morphologies, respectively. 

## 2. Materials and Methods 

### 2.1. Chemicals

All of the reagents used for the synthesis of polymer matrices and for the surface modification of nanoparticles were purified by specific procedures, since they were not commercially available under the highest purity. These purification methods and detailed handling of anionic polymerization synthesis procedures, together with the high-vacuum technique, are reported in detail elsewhere [38,39].

### 2.2. Instrumentation

Structural characterization was performed through atomic force microscopy at room temperature. The atomic force microscopy (AFM) images were collected in tapping mode (TM-AFM) with a Bruker Multimode 3D Nanoscope using a microfabricated silicon cantilever type TAP-300G, with a tip radius of <10 nm and force constant of approximately 20–75 N·m^−1^. High resolution transmission electron microscopy (HR-TEM) measurements were carried out in a JEOL 2100 TEM by using 200 KeV as acceleration voltage and a CCD camera to analyze the particle size and shape. Toluene diluted batch of γ-Fe_2_O_3_ particles was prepared with a concentration of 1% *w*/*v* and, subsequently, a small drop of the solution was placed onto 600 mesh carbon coated Cu grids for TEM analysis (drop cast method). Evaporation of the solvent is needed (approximately 3–5 days under ambient conditions) and the grid is dried under vacuum for at least 2 h in order to successfully investigate the sample. The number and weight average molecular weights and dispersity (*Ð*) of the BCP hosts and homopolymer (hPS) were determined via size exclusion chromatography (SEC). The SEC instrument was equipped with an isocratic pump (SpectraSystem P1000), column oven (Lab Alliance) heated at 30 °C, one pre-column (PLgel 5μm, Guard, 50 × 7.5 mm), three columns in series (PLgel 5 μm Mixed-C, 300 × 7.5 mm), refractive index detector (RI, Shodex RI-101), and two-angle laser light scattering (TALLS) detector. The number-average molecular weights (values higher than 15,000 g/mol) were measured with a Gonotec membrane osmometer Osmomat 090 at 35 °C. Toluene, dried with CaH_2_, was used as the solvent for the measurements.

The TGA measurements were conducted in a Perkiln-Elmer (Pyris Diamond) thermal analyzer under nitrogen flow by studying the heating circle ranging from 10 °C to 800 °C with the applied heating rate at 278 K/min. to separately measure the organic and inorganic portion via the weight loss amount (% wt). The infrared measurements were recorded by FT-IR spectrometer (FT-IR 8400, Shimadzu Corporation) in the range from 4000 to 400 cm^−1^ at room temperature. NPs (neat and functionalized with low molecular weight PS chains) were diluted into 4% *w*/*v* toluene solution, mixed with potassium bromide tablets, and, after drying, were placed to the FT-IR stage.

#### 2.2.1. Block Copolymer Synthesis

**Scheme 1:**
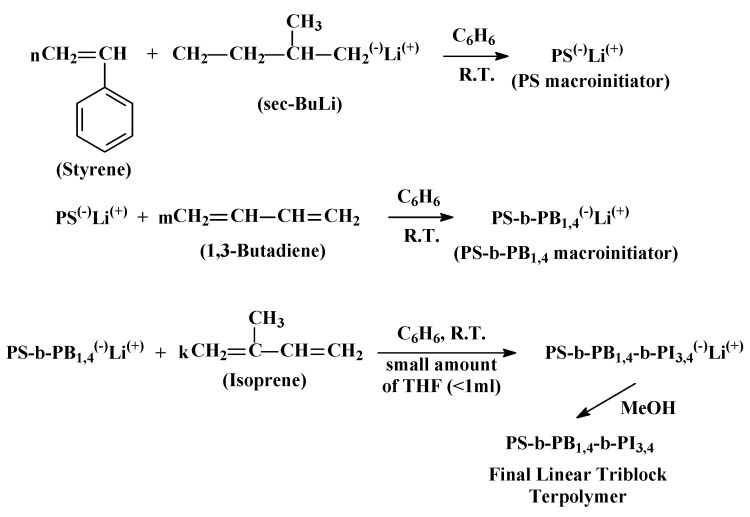
Synthesis reactions for the preparation of linear triblock terpolymer of the required PS-*b*-PB_1,4_-*b*-PI_3,4_ sequence.

The synthesis of the PS_25_-*b*-PB_23_-*b*-PI_12_ type linear triblock terpolymer was accomplished by sequential anionic polymerization of styrene (4.0 g, 38.46 mmol), butadiene (3.6 g, 66.67 mmol), and isoprene (1.9 g, 27.94 mmol) in benzene, while using sec-BuLi as initiator (0.158 mmol). After the polymerization of styrene in benzene (300 mL) in room temperature conditions, an appropriate amount of butadiene is distilled in the apparatus to successfully prepare the necessary intermediate block PB of the PS_25_-*b*-PB_23_-*b*-PI_12_ linear terpolymer sequence. Prior to the addition of the third monomer (isoprene), a small quantity of THF(1 mL) is inserted in the reactor to improve the kinetics of the living ends and gain the favorable 3,4- microstructure (~55–60%) for the PI segments. It should be mentioned that the microstructure of the PB block is very high (~92%) in −1.4 microstructure, since it has been synthesized in non-polar environment. Accordingly, the second sample of the PS_45_-*b*-PB_34_-*b*-PI_74_ sequence is being prepared, but with different quantities of the monomer and the initiator. The microstructures of the polydienes are similar (as calculated by proton nuclear magnetic resonance spectroscopy, ^1^H-NMR spectroscopy). Full detailed synthesis of the terpolymers is described in the literature [35,36,37]. Scheme 1 shows the reactions for the synthesis of the triblock terpolymers. The PS-*b*-PB diblock copolymers were similarly synthesized, as described above, exclusively in benzene. 

#### 2.2.2. Polystyrene Grafted γ-Fe_2_O_3_ Nanoparticles Functionalization

**Scheme 2:**
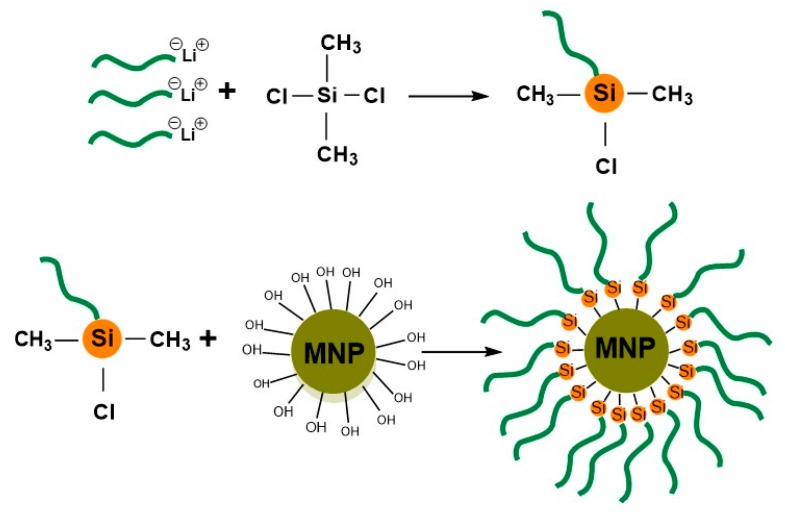
Two step reaction leading to the functionalization of the magnetic nanoparticles (NPs) surface by the modified with the suitable chlorosilane [(CH_3_)_2_SiCl_2_] PS chains.

Maghemite iron oxide nanoparticles (IONPs) that were approximately 10–11 nm in diameter were synthesized following the Massart’s coprecipitation method [40,41]. Exploiting the chlorosilane-linking chemistry as previously reported by our group [42], magnetic nanoparticles were functionalized by the “grafting to” method of homopolymer hPS chains (Mn = 6.5 Kg/mol). In this approach, the homopolymer bears functional groups (chlorine atoms) due to its linking reaction with dichlorodimethylsilane [(CH_3_)_2_SiCl_2_]) (Scheme 2). The use of high excess (150:1) of (CH_3_)_2_SiCl_2_ in respect to living PS enabled the substitution of only one chlorine atom and the formation of PS-Si(CH_3_)_2_Cl, as indicated on the IR spectrum (supplementary files, Figure S1). All of the reaction steps were conducted in benzene and the excessive amount of (CH_3_)_2_SiCl_2_ and the solvent were removed in the high vacuum line through the liquid nitrogen trap.

#### 2.2.3. Preparation of IONPs/BCP Composite Films

The samples for AFM measurements were prepared by diluting the BCPs and IONPs in toluene (~ 1% *w*/*v* ). The mixtures were sonicated for a few minutes and subsequently stirred for approximately one day to adopt optically transparent and homogeneously dispersed solution. Polymer films 30–45 nm thick (ellipsometry) were spin-coated onto pre-cleaned silicon wafers in a “piranha” solution (a mixture of concentrated sulfuric acid (70 vol%) and hydrogen peroxide solution (30 vol%)) at 80 °C for 1 h to oxidize the surface and, finally, the wafers rinsed with water and ethanol and then dried. Specifically, a few drops from each solution were deposited onto the spin coater stage rotating at approximately 3000 rpm for 30 sec. in order to evaporate the excessive amount of solvent.

## 3. Results and Discussion 

The high-vacuum anionic polymerization technique was utilized to synthesize the diblock copolymers (di/BCP) and triblock terpolymer (tri/BCP), which were examined as topographical nanopatterns with iron oxide nanoparticles (Table 1). The investigation is mainly focused on the required time for the BCP/NPs system to reach the phase equilibrium and how the addition of the third block (going from AB to ABC BCPs) affects the respective time and the final morphology. Spin coating permits the homogeneous deposition of the sample onto the silicon wafer substrate optimizing the parameters that are related to spin speed, and solution concentration. 

**Scheme 3:**
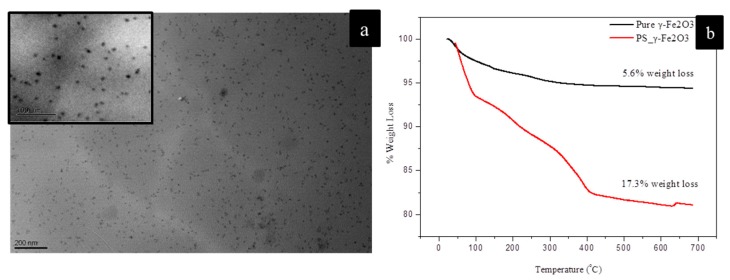
(**a**). PS-functionalized nanoparticles as indicated from Transmission Electron Micrsoscopy image following the drop casting method (Scale bar 200 nm) (**b**). TGA graphs of pure and grafted particle batch onto their periphery with PS chains.

For comparison reasons, the surface structures of the neat di/BCP and tri/BCP hosts were equally examined by TP-AFM (Figures S2–S4). It should be mentioned that, for all polymer samples, the molecular characteristics were chosen to adopt lamellar morphologies during self-assembly when studied with transmission electron microscopy. More specifically, alternating lamellae was evident for both diblock copolymers (Figures S3 and S4) and three-phase four-layer lamellae was identified for both triblock terpolymer systems (Figure S5), respectively. The decoration of particle surface with the PS layer, was studied with the use of TEM images (Scheme 3a) and TGA thermographs (Scheme 3b). The value calculated for the grafting density (0.17 chains nm^−2^) proved to be sufficient to generate a mutual affinity between functionalized IONPs and the PS domains. The grafting density value was calculated from the weight loss of TGA thermograph (Scheme 3b), as reported previously [42].

**Figure 1:**
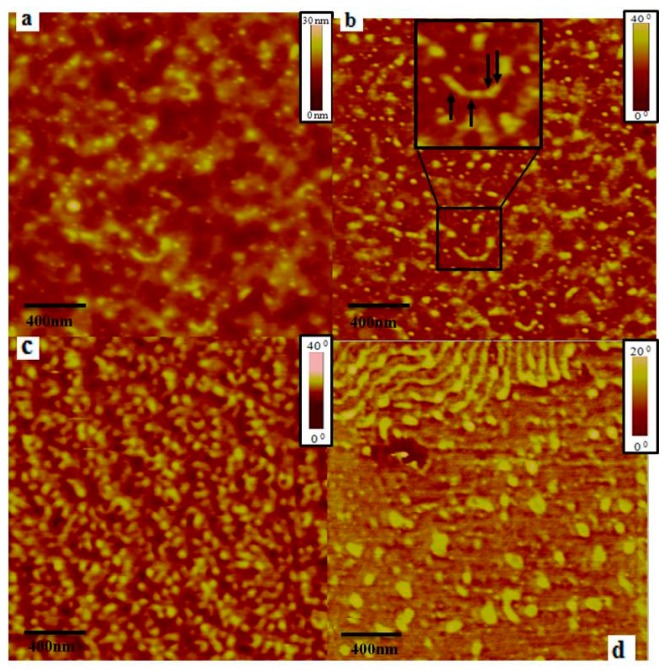
Height and phase (**a**,**b**) images after solvent vapor annealing (SVA) treatment for 90 min. of PB_40_-*b*-PS_40__PS-Fe_2_O_3_ composite thin film. In Figure 1b, an enlarged view is given with arrows indicating the position of the NPs. Phase image (**c**) correspond to SVA treated film for 6 h. Phase image (**d**) depicts the morphology for 24 h SVA treatment. The NPs loading is kept constant at 10% wt. All samples correspond to almost identical film thickness.

Images of (12 ± 2 nm) PS-grafted γ-Fe_2_O_3_ IONPs with PB_40_-*b*-PS_40_ that were dispersed in toluene solution (an almost good solvent for both PB and PS) and NPs concentration 10% wt were initially studied. For the as-spun state, a slightly ordered lamellar-like morphology is shown (Figure S6). Bright spots are considered to be PS-grafted IONPs, since the IONPs are much harder than polymer chains. These spots tend to occupy both yellow (PS domains) and dark phases (PB domains), indicating no spatial selectivity for the NPs. The same film was annealed in saturated toluene vapors under autoclave conditions at constant temperature (30 °C) for different duration times to observe the structure upon solvent vapor annealing (SVA) of the IONPs/BCP composite. For the solvent vapor annealing experiments, the essential adjustment of a glass-formed reactor to an autoclaved chamber of identical capacity (250 mL volume) was used (Figure S7). Primarily spin-coated films were placed for various periods of time into the chamber and they were mounted on a glass flat surface. Autoclave conditions are achieved by adjusting a metallic ring and clamp, around the lid of chamber. Except for the typical constant temperature in all cases (~30 °C), no further control of the solvent vapor pressure could be performed. We decided for this study to keep the SVA temperature well below the T_g_ of the PS domains and investigate NPs incorporation based on the duration of the SVA at conditions close to ambient ones. The temperature of 30 °C was good enough to slowly create representative solvent vapors under autoclave conditions. The adopted surface structure of the hybrid film under several SVA duration times (1.5 h, 6 h, and 24 h) revealed significant information regarding the particle localization and the resulting BCP self-assembly. Unlike the as-spun film, individual NPs were distributed within the PS domains (Figure 1a,b), while the agglomerated ones tend to overwhelm the “guideline” boundaries of PS block. The majority of IONPs are segregated within the brighter stripes, corresponding to the PS domain, as better indicated from the phase image (Figure 1c).

Upon increasing the SVA time to 6 h (Figure 1c) tortuous and circular features were both formed by the PS domains. The phase separation of the structure is perturbed by the large IONPs aggregates. Nearly all of the IONPs have been embedded within the brighter stripes, but the relatively larger size of the yellow spots displays the tendency for agglomeration, leading to irregular grains. Inevitably, these aggregates are not sequestered within the narrow stripes of PS domains, but they tend to unsettle the boundaries of the lamellae structure. After 24 h of SVA treatment (Figure 1d), a significant portion of captured surface shows NPs aggregates within the boundaries of PS stripes, but not for the entire surface, leading to relatively weak long-range order film formation. The local variation of the lamellae width can be attributed to the assembly of NPs inside the PS domain. The different disposition and direction of the alternating lamellae features is another indication that confirms the selective incorporation of PS modified IONPs within the PS layers. The spatial embedding of individual NPs or their aggregates can be illustrated by the comparable width of the PS stripes, since the larger aggregates in size led to the swelling of the spatial lamellae thickness [43]. Finally, the collapse of the structure is evident in areas where even larger aggregated clusters of NPs were present. 

**Figure 2:**
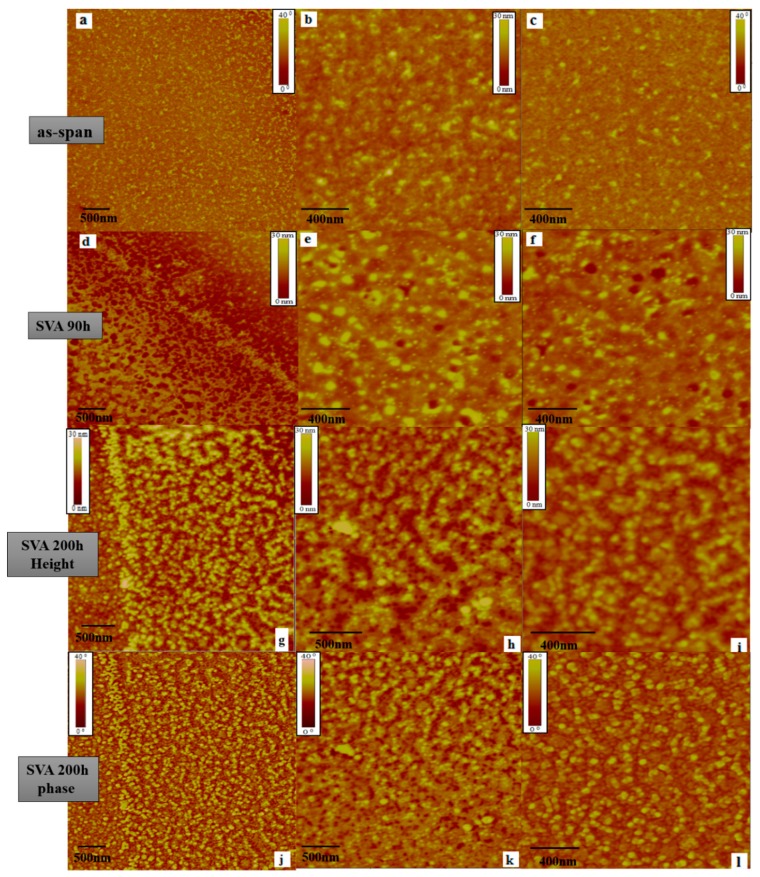
TP-AFM images describe the morphological evolution under SVA treatment of PS_50_-b-PB_50__PS-γ-Fe_2_O_3_ composite. For as-spun state, height and phase (**a,b**) as well as a closer phase views (**c**) are given. Height (**d**) and phase (**e**) as well as closer phase views (**f**) after 90 h are also depicted. The SVA treatment finally induced morphology is also shown for two different scanned areas height (**g,h**) and phase (**j,k**) for 200 h respectively. Magnified height (**i**) and phase (**l**) views of a specific area (from initial area of **h, k** views) after 200 h of SVA treatment. The NPs loading is kept constant at 10% wt.

We used a higher molecular weight diblock copolymer with a similar volume fraction in order to verify these results (PS_50_-*b*-PB_50_ instead of PB_40_-*b*-PS_40_ as mentioned above). The nanocomposite film of γ-Fe_2_O_3_ particles that were mixed with the higher molecular weight BCP was similarly studied with TP-AFM, which verified a slightly different tendency for phase segregation. Regarding SVA duration time, significantly longer times are required to observe perpendicularly oriented microdomains where NPs selectively occupy only one of the two phases. In the first series of images (Figure 2a,c (phase images) and Figure 2b (height image)) the as-spun film (without any SVA treatment) provides no evidence of microphase separation. SVA treatment for 90 h reveals the coexistence of individual NPs and small size NPs aggregates randomly placed within the domains, as shown in Figure 2d–f. Due to lack of phase contrast between adjacent blocks, it is impossible to verify their distribution. The texture of homogeneous NPs deposition on the top of the thin film is evident, probably due to the chain relaxation of both blocks. Additionally, an early stage of microphase separation is also shown. Such a thermodynamically driven tendency is validated by the topography (height) of two different areas of the film surface (Figure 2e,f).

Increasing the SVA duration time from 90 to 200 h leads to a well-ordered structure. It is evident that the NPs arrangement is still poor despite this well-oriented structure formation, probably due to the formation of aggregates. Conversely, a large area of the same image indicates a great affinity of PS-grafted IONPs with the PS domain, eventually leading to the swelling of the PS stripes. Phase images permit more distinct analysis of the topographical features. A phase image highlights the edges of particular stripes, since it is not affected from large scale height deviations, due to the increased surface roughness [44]. This is the reason that our study is mostly based on the observation of phase images that can visualize the phase contrast between the adjacent blocks when compared to the corresponding height image. In summary, individual IONPs and small aggregates are both selectively localized within alternating perpendicular lamellae of di/BCP substrate at 200 h, while, by additional SVA duration, the film structure remains unaffected. 

Our morphological study was further extended by adding a third chemically different block with significantly high 3,4-microstructure (in order to obtain a three-phase system as already reported in the literature [35]). The conditions for the SVA experiments were similar to those that were used for the diblocks (30 °C at different duration times), with the only difference being the alternation of the solvent from toluene to benzene. Benzene was preferentially used due to the non-selectivity as a good dilution media for all three different segments, as already reported in the literature [35]. As a function of the SVA time, the surface topography of the generated ABC terpolymer (sample PS_45_-*b*-PB_34_-*b*-PI_74_) based composite was also analyzed likewise to the aforementioned diblock nanocomposites. For the neat ABC terpolymer, a perpendicular morphology leads to a clear observation of two and not three components in the TM-AFM images (Figure S4). The absence of the third phase is attributed to common viscoelastic properties of both polydiene blocks, which are detected by the scanner tip as one phase, regardless of the film thickness. 

**Figure 3:**
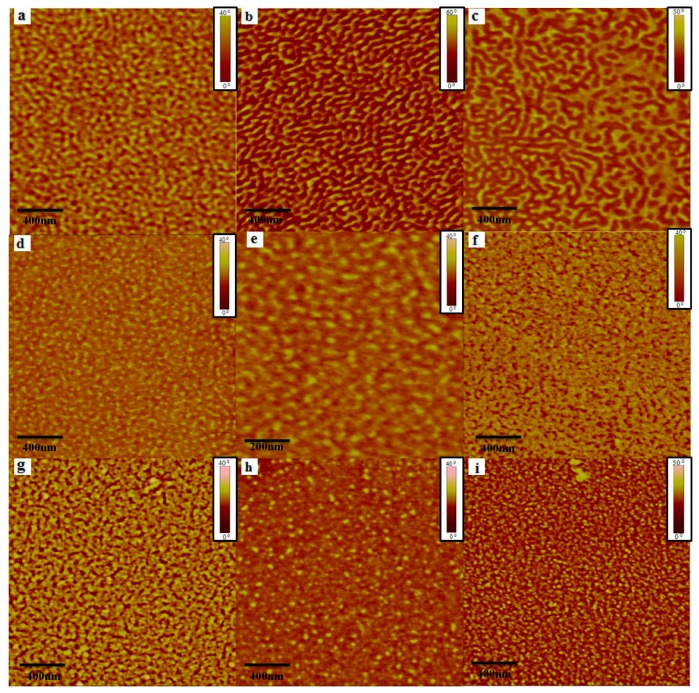
Phase images gallery of as-spun and after specific time duration for SVA treatment of sample PS_45_-*b*-PB_34_-*b*-PI_74_/PS_grafted γ-Fe_2_O_3_ (10% wt.). The time of treatment in benzene vapors was: (**a**) as-spun, (**b**) 2 h, (**c**) 4 h, (**d**) 18 h (**e**) 18 h (closer view), (**f**) 60 h, (**g**) 96 h, (**h**) 120 h, and (**i**) 192 h. Scan size for all phase images is at 2 μm × 2 μm, except from image, (**e**) which corresponds to higher magnification (1 μm × 1 μm).

Even though the morphological behavior of the tri/BCP hybrid film is more complex when compared to the di/BCP cases, the directed self-assembly of IONPs into chemically similar domain of PS appears to gradually improve upon the increase of SVA duration time under the same temperature conditions in benzene instead of toluene (as in the di/BCP cases). As for di/BCPs IONPs in the AFM images, bright areas correspond to the hard PS phase, while dark areas to the polydienes due to the thermodynamically driven phase separation [45]. Taking the physical size of the NPs into account, it is clear that bright spots do not correspond to only individual particles, but also to small aggregates [46]. At the as-spun image (Figure 3a), the surface pattern comprises of uniformly microphase separated domains, while the IONPs cannot be distinguishable from the underlying stripes texture. Almost the same description fits with the next two images (Figure 3b,c) corresponding to results from SVA times for 2 and 4 h, respectively. The stripes of PS domains are shown more elongated in the 2 h case, whereas after 4h the same domains seem to acquire larger width and length.

The first sign of typically organized and well-distributed NPs is observed after 18 h of SVA treatment (Figure 3d,e). In Figure 3e, although the good dispersion of the NPs is obvious, there is no particular affinity of sequestration for each domain. The AFM images for 60 h SVA (Figure 3f) and for 96 h (Figure 3g) emphasize an increase in the phase contrast between thermoplastic (PS) and elastomer (PB, PI) domains and the hard texture of the NPs seemingly attributed to NPs aggregates located within PS domain. 

The images corresponding to 120 h (Figure 3h) and 192 h (Figure 3i) of SVA treatment only indicate the arrangement of individual NPs and small size agglomerations. Interestingly enough, below this inorganic texture, as formed from the IONPs, someone might faintly observe the underlying microphase separated pattern. Analogous imaging features have been reported in the literature from Garcia, Mondragon et al. by sequestering PS grafted magnetic NPs in specific concentration (4% wt) [16].

**Figure 4:**
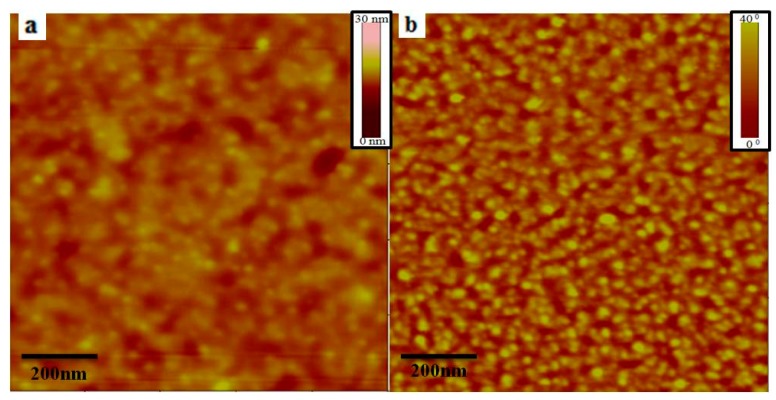
TM-AFM height (**a**) and phase (**b**) images of sample PS_45_-*b*-PB_34_-*b*-PI_74_/PS_grafted γ-Fe_2_O_3_ (10% wt.) corresponding to the 192 h SVA treated composite film.

In contrast with the di/BCP hosts, the time efficiency in the tri/BCP solvent annealing process plays a more prominent role [47]. The film formation process can be divided in two stages. Firstly, the nanoparticles emerge on the top of the surface (for the first 96 h) and then the swelling of the blocks takes place due to the chemical affinity of the PS-grafted NPs with the PS domain. As already mentioned for toluene, benzene also exhibits a slight selectivity towards PS. Such a mutual affinity provokes the polymer mobility in the swollen state due to solubility constrains. Consequently, this structural rearrangement facilitates the NPs to migrate within chemically mutual domains. Therefore, the vast majority of ΙOΝPs appear to be either individually placed or slightly agglomerated (Figure 4). The underlying phase segregated pattern is described by the combination of a discontinuous lamellae. By increasing the SVA time, no indication of improved surface structure is observed.

**Figure 5:**
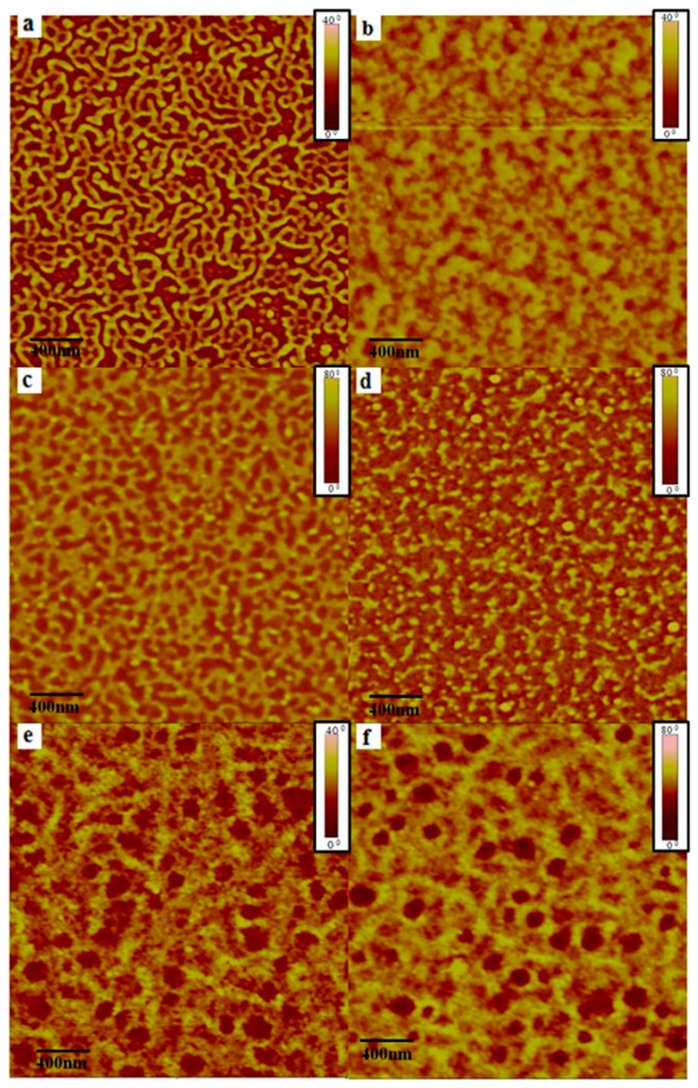
Phase images of thin films for sample PS_26_-*b*-PB_23_-*b*-PI_12/_PS_grafted γ-Fe_2_O_3_ (10% wt.) (**a**) before exposure in benzene vapors, (**b**) after 5 h, (**c**) 25 h, (**d**) 50 h, (**e**) 72 h, and (**f**) 96 h. Scan size: 2 × 2 μm.

The self-assembled nanostructures of the PS_25_-*b*-PB_23_-*b*-PI_12_ terpolymer host (which is a lower total molecular weight triblock terpolymer when compared to PS_45_-*b*-PB_34_-*b*-PI_74_) comprising of similar block sequence and percentage of filler (10% wt PS_grafted γ-Fe_2_O_3_) was also investigated. The as-spun film (Figure 5a) for the corresponding surface hybrid pattern indicates the irregular formation of bright continuous lines or circular spots of the PS domain phase. The effect of SVA duration becomes apparent after the first 5 h in benzene vapors, where the swelling of the PS domain is attributed (Figure 5b) to the slight selectivity of the solvent vapors (benzene) towards PS chains. This morphological effect causes more intensive changes in the effective volume of the domains in the corresponding triblock terpolymer. The lower total molecular weight (and lower molecular weight per block) of this host drives the system to constantly induce variable features through the thermodynamically driven process of annealing. Regardless of this morphological change, particles are still not evident on the top of the scanned surface. By such a low molecular weight tri/BCP matrix, we assume that it is difficult to control the particle sequestration, due to the lack of available space of the PS domain. This fact suggests weaker phase separation when compared to PS_45_-*b*-PB_34_-*b*-PI_74_, not allowing the tri/BCP lamellae platform to adopt narrow phases and long–range order structures. By further extending the SVA duration, the most fascinating results were obtained after 72 h of SVA treatment (Figure 5e). In this case, the induced surface topology adopts the structure of dark holes that are surrounded by PS coils. 

Inevitably, this structural difference results in variable domain periodicity (L_0_), as depicted from the TEM images of the corresponding neat tri/BCP sample (Figure S5). Upon incorporating the same batch and volume fraction of PS_γ-Fe_2_O_3_ particles, which means that the dimension and concentration remains constant (10% wt), the size of the PS lamellae (11 nm) is not sufficiently large to host the nanoparticles in the PS_25_-*b*-PB_23_-*b*-PI_12_ sample. For the same reason upon embedding the same batch of IONPs into the PS_45_-*b*-PB_34_-*b*-PI_74_ tri/BCP sample, the domain size of PS is much larger (40 nm) and enables the NPs deposition within the PS domain more effectively. 

Bright spots are localized on the PS domain, as it is evident in Figure 5c,d. SVA after 72 and 96h (Figure 5e,f) indicates that these spots do not have a size that is comparable with the NPs size, as calculated by the TEM analysis (Scheme 3a), and the most plausible explanation is the NPs formed agglomerates. Although the NPs exhibit great affinity with the PS regions, the high load of particles, in addition to the lower segment size, prohibits any preferential incorporation within the PS domain. Even though the composite film surface appears to progressively being covered with particle aggregations (Figure 5e,f) or random clusters, the empty space of dark regions, free of NPs, is clearly obvious [48].

## 4. Conclusions

We present the effect of the SVA process on the developed morphology by the addition of one extra block in a BCP patterned platform based on morphological results that have been obtained so far (from AB to ABC), which has never been previously reported in the literature. The PS-grafted maghemide particles spatial localization within PS domains was driven by the self-assembly of di/BCPs and tri/BCPs. The chemical affinity and the SVA treatment by avoiding the use of selective solvent for any segment were effective toward the controlled confinement of IONPs within the PS domain of either di/BCPs or tri/BCPs. The addition of PI component of specific geometric isomerism to microphase separate from PB domains strengthens the suggestion that lowering the molecular weight of the BCP host is a viable route for improving the order of the finally adopted structure. However, the molecular weight decrease can limit sufficient domain spacing for particle confinement within a specific domain, as was found for the low molecular weight PS_25_-*b*-PB_23_-b-PI_12_ terpolymer. The chemical modification of the particle surface with PS chains enabled the selective incorporation of IONPs on higher molecular weight tri/BCPs, while the same particle batch led to aggregated clusters on top of the film surface for significantly lower molecular weight matrix of tri/BCPs. The SVA method was found to be an efficient method of selective NPs incorporation on to the PS domains for the lamellar structured ABC BCP host. After trials and errors, the percolation threshold was estimated roughly as 10% in volume (10% *w*/*v*), since higher IONPs load (for example 12.5% *w*/*v*) led to the “collapse” of the microstructure, as is evident in Figure S8.

## Figures and Tables

**Table 1:** Molecular characteristics of diblock copolymers and triblock copolymers that were used as block copolymer (BCPs) matrices.

Samples	$({\bar{M}}_{n}^{PS}{)}^{\alpha }$ (Κg/mol)	$({\bar{M}}_{n}^{PB}{)}^{\alpha }$ (Κg/mol)	$({\bar{M}}_{n}^{PI}{)}^{\alpha }$ (Κg/mol)	$({\bar{M}}_{n}^{\mathrm{total}}{)}^{a}$ (Κg/mol)	*Ð ^b^*	f_PS_^c^	f_PB_^c^	f_PI_^c^	N_PS_/N_PB_/N_PI_^d^
PS_40_-*b*-PB _40_	40.5	41.3	-	81.8	1.07	0.50	0.50	-	389/765/-
PS_50_-*b*-PB_50_	48.2	50.6	-	98.8	1.08	0.49	0.51	-	463/937/-
PS_45_-*b*-PB_34_-*b*-PI_74_	45.0	34.2	74.1	153.3	1.09	0.30	0.21	0.49	433/633/1090
PS_26_-*b*-PB_23_-*b*-PI_12_	25.8	23.4	12.2	61.4	1.10	0.43	0.37	0.20	248/433/179

^a^ Membrane Osmometry in toluene at 35 °C. ^b^ SEC in THF at 30 °C. ^c^ ^1^H-NMR in CDCl_3_ at 25 °C. ^d^ Degrees of polymerization for the different segments based on the number average molecular weights and the monomer molecular weight.

## Supplementary Materials

The following are available online at https://www.mdpi.com/1996-1944/13/6/1286/s1, Figure S1: Infrared spectra of γ-Fe_2_O_3_ magnetic nanoparticles (indicated with red) and γ-Fe_2_O_3_ magnetic nanoparticles functionalized with polystyrene chains (indicated with black). Figure S2: AFM and TEM images for PB_40_-b-PS_40_ after proper treatment for thin and bulk films separately. Images a,b show the height and phase of the produced morphology after 6 h exposure in toluene vapors at scan size: 2 × 2 μm respectively. At image c, larger scanned area of scan size 4 × 4 μm is shown. Image d depicts the cross section TEM image after 5 days thermal annealing at 115 °C followed by ultramicrotomy treatment. Scale bar: 200 nm. Figure S3: AFM and TEM images for PB_50_-*b*-PS_50_ after proper treatment for thin and bulk films separately. Images a,b presents the height and phase of the produced morphology after 6 h exposure in toluene vapors respectively at scan size: 2 × 2 μm. In image c a properly conditioned film at scan size 4 × 4 μm is given. Image d depicts the cross section TEM image after 5 days thermal annealing at 115 °C followed by ultramicrotomy treatment. Scale bar: 100 nm. Figure S4: TP-AFM height (left) and phase (right) images for the neat samples of PS_25_-*b*-PB_23_-*b*-PI_12_ (a,b) and PS_45_-*b*-PB_34_-*b*-PI_74_ (c,d) respectively after 24 h annealing in benzene vapors inside the autoclave chamber (solvent vapor annealing). Figure S5: TEM images for the two linear triblock terpolymers of different total molecular weight indicating the PS-*b*-PB-*b*-PI_3,4_ sequence. Image a corresponds to PS_45_-*b*-PB_34_-*b*-PI_74_ and image b to PS_25_-*b*-PB_23_-*b*-PI_12_ respectively. The indices next to the blocks in both samples indicate the number average molecular weight of each block respectively. Figure S6: TP-AFM height (a,c) and phase (b,d) images for as-spun PS_40_-*b*-PB_40__PS-γ-Fe_2_O_3_ composite thin film. Two different scan size of 4 × 4 & 2 × 2 μm (enlarged) views depict the NPs arrangement with loading rate 10% wt. Figure S7: Side (a) and top (b) view of the improvised apparatus for the solvent vapor annealing procedure. Within this autoclave chamber, a glass substrate is evident up on which the Si wafers are deposited beneath which the solvent resides which stands on top of solvent deposit in a specific smaller chamber. Figure S8: TP-AFM height (a) and phase (b) images for PS40-*b*-PB40_PS-γ-Fe_2_O_3_ composite thin film with loading rate 12.5% wt after 24 h SVA.
