# Supplementary material for: Segregation of Maghemite Nanoparticles within Symmetric Diblock Copolymer and Triblock Terpolymer Patterns under Solvent Vapor Annealing
Source: Materials (Basel). 2020 Mar 12;13(6):1286. doi: 10.3390/ma13061286 (PMC7142801; doi:10.3390/ma13061286)
Supplement: Supplementary file 1 [file materials-13-01286-s001.pdf]

materials

Supplementary Materials

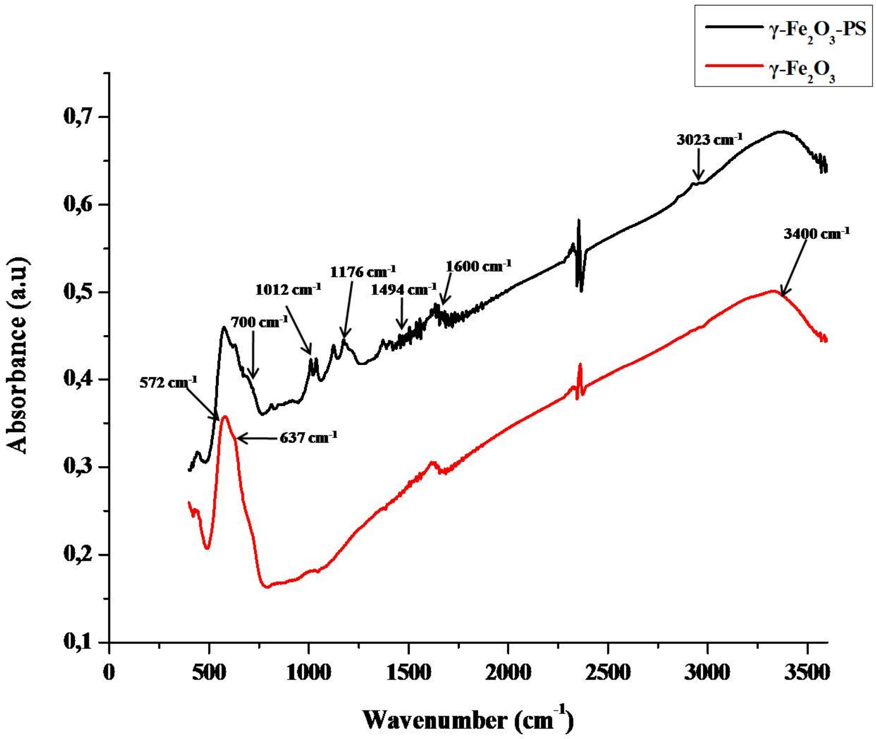

Figure 1. Infrared spectra ofγ-Fe_2O_3 magnetic nanoparticles (indicated with red) and γ-Fe_2O_3 magnetic nanoparticles functionalized with polystyrene chains (indicated with black).

The FTIR spectrum of neat γ-Fe_2O_3 nanoparticles exhibits three characteristic peaks at 3400 cm^{-1}, 637 cm^{-1}, and 572^{-1} cm, attributed to the hydroxyl groups on the nanoparticles surface, the Fe-O-Fe and Fe-OH bonds, respectively. The functional nanoparticles with PS chainsγ-Fe2O3-PS exhibits the characteristicpeaks at Si-O-Fe at 1176 and 1012 cm^{-1}, C-H aromatic stretching at 3023 cm^{-1}, C-C stretching frequency of the ring at 1600 cm^{-1}, C-C stretching vibration of the ring at 1494 cm^{-1} andC-H out of plane bending vibration of the ring at 700 cm^{-1}.

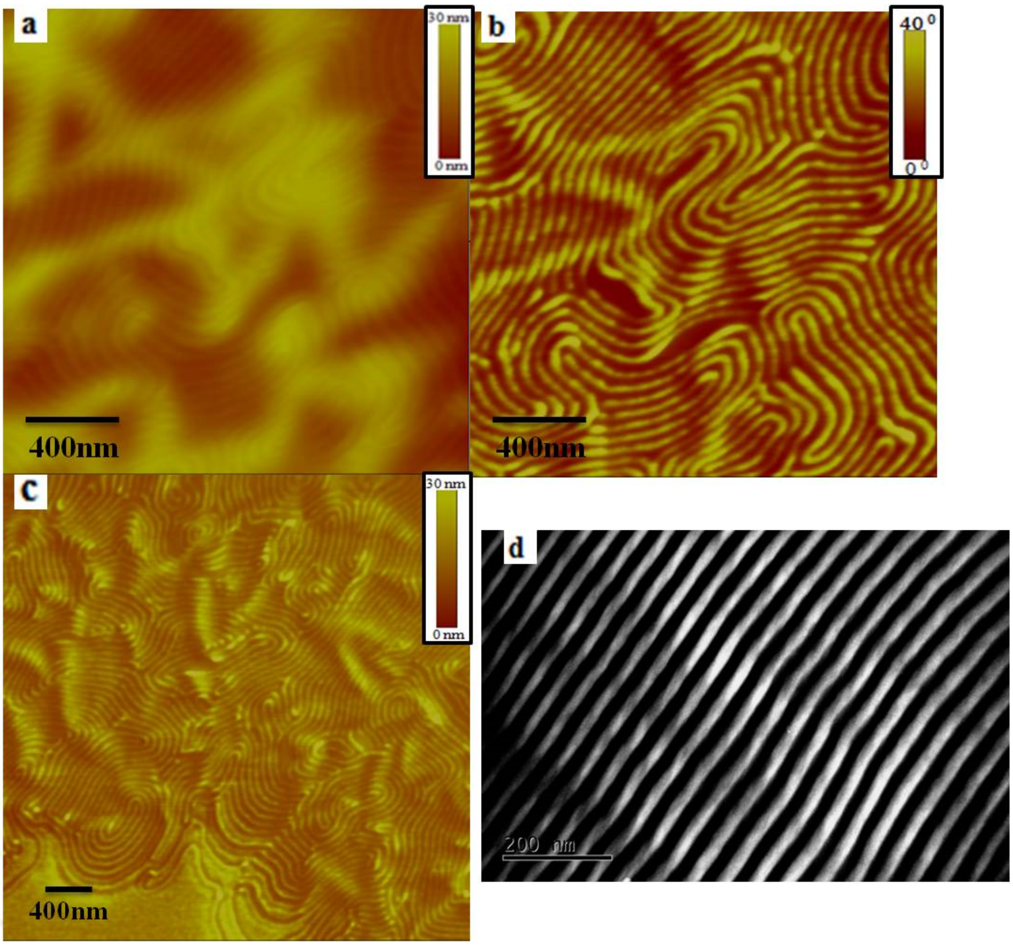

Figure 2. AFM and TEM images for PS_{40}-b-PB_{40} after proper treatment for thin and bulk films separately. Images a and bshow the height and phase of the produced morphology after 6 h exposure in toluene vapors at scan size: 2 μm x 2 μm respectively. At image c, larger scanned area ofscan size 4 μm x 4 μm is shown. Image d depicts the cross section TEM image after 5 days thermal annealing at 115 °C followed by ultramicrotomy treatment. Scale bar: 200 nm.

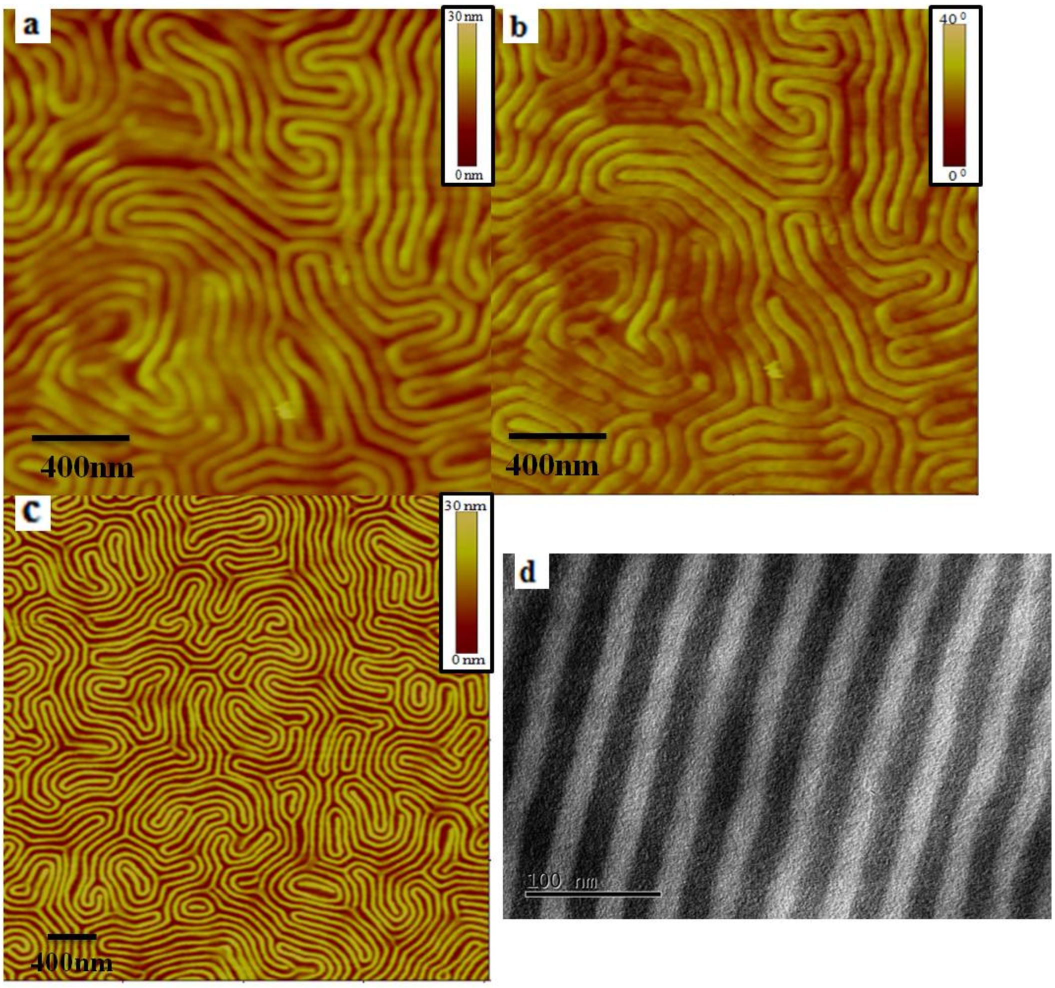

Figure 3. AFM and TEM images for PS_{50}-b-PB_{50} after proper treatment for thin and bulk films separately. Images a,b presentsthe height and phase of the produced morphology after 6 hour exposure in toluene vapors respectively at scan size: 2 μm x 2 μm. In image c a properly conditioned film at scan size 4 μm x 4 μm is given. Image d depicts the cross section TEM image after 5 days thermal annealing at 115 ^{0}C followed by ultramicrotomy treatment. Scale bar: 100 nm.

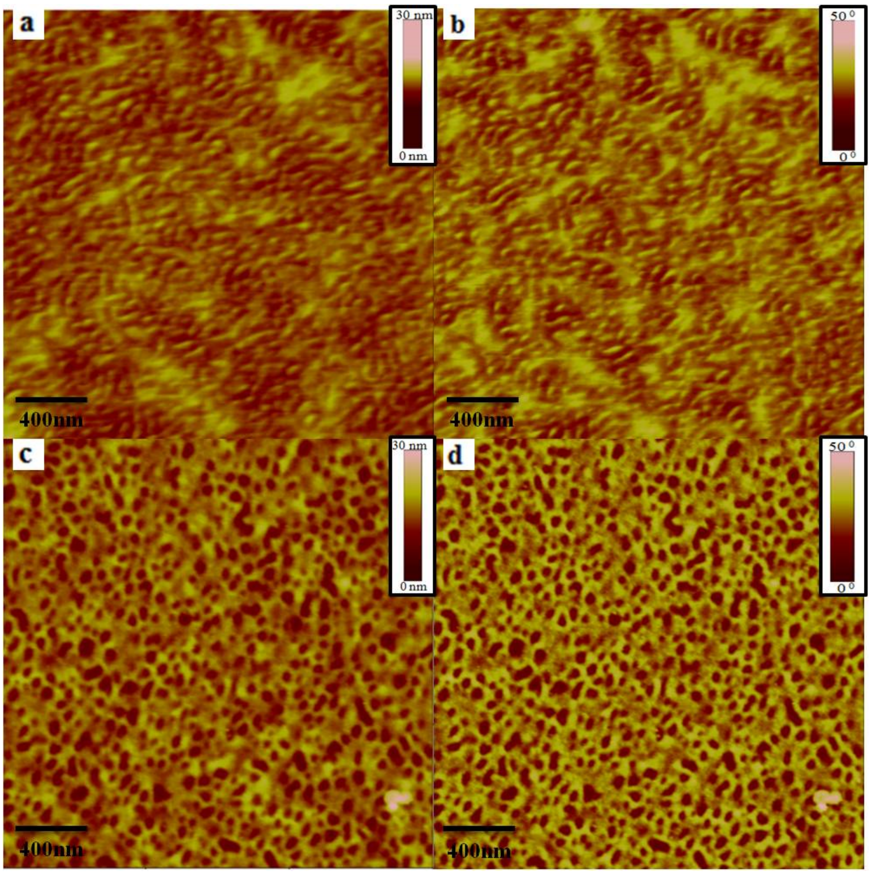

Figure 4. TP-AFM height (**left**) and phase (**right**) images for the neat samples of PS_{25}-b-PB_{23}-b-PI_{12} (**a,b**)and PS_{45}-b-PB_{34}-b-PI_{74} **(c,d)** respectively after 24 hours annealing in benzene vapors inside the autoclave chamber (solvent vapor annealing).

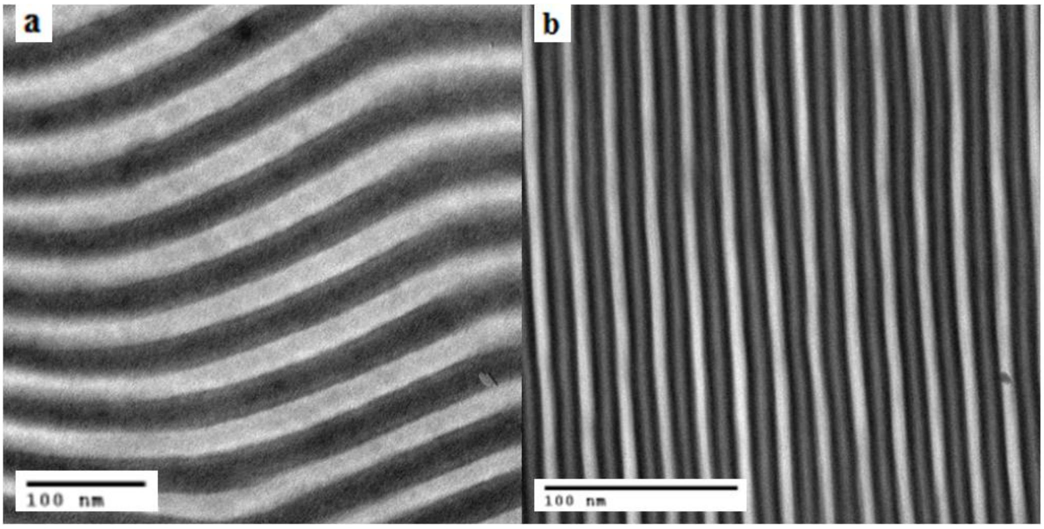

Figure 5. TEM images for the two linear triblock terpolymers of different total molecular weight indicating the PS-b-PB-b-$PI_{3,4}$ sequence. Image (**a**) corresponds to PS_{45}-b-PB_{34}-b-PI_{74} and image (**b**) to PS_{25}-b-PB_{23}-b-PI_{12} respectively. The indices next to the blocks in both samples indicate the number average molecular weight of each block respectively.

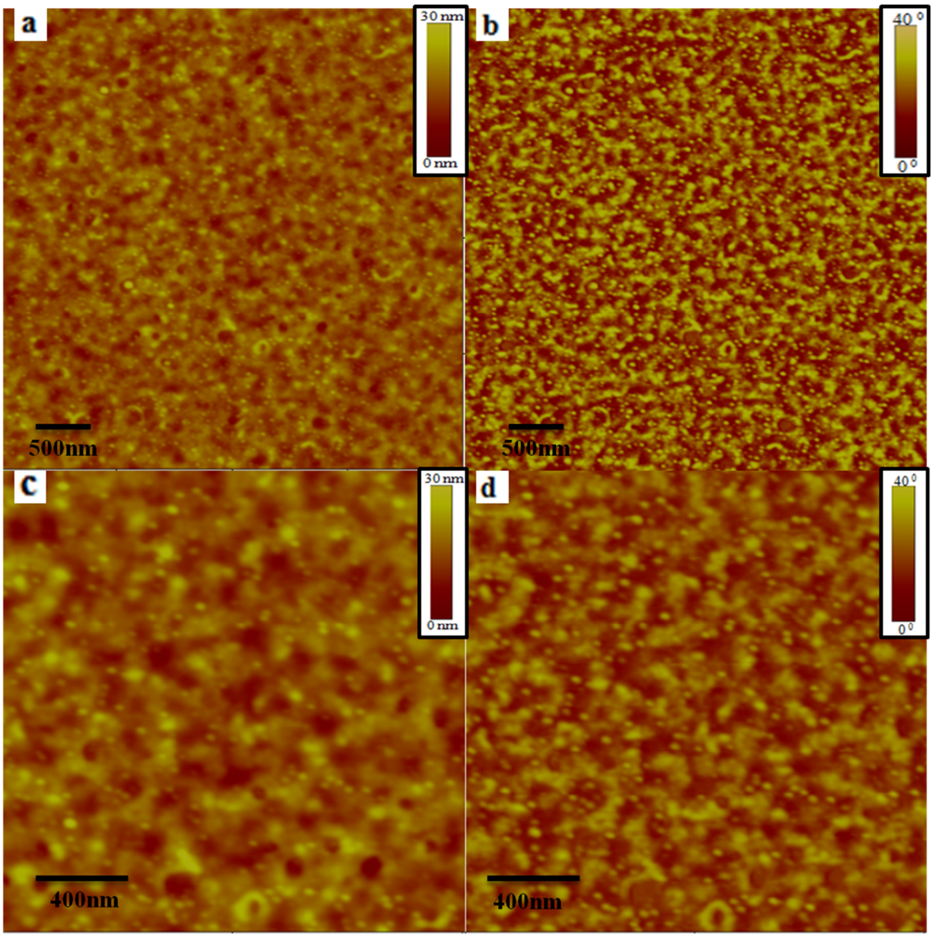

Figure 6. TP-AFM height (**a**,**c**) and phase (**b**,**d**) images for as-spun PS_{40}-b-PB_{40}_PS-γ-Fe_2O_3 composite thin film. Two different scan size of **4 μm x 4 μm & 2 μm x 2 μm**(enlarged) views depict the NPs arrangement with loading rate 10% wt.

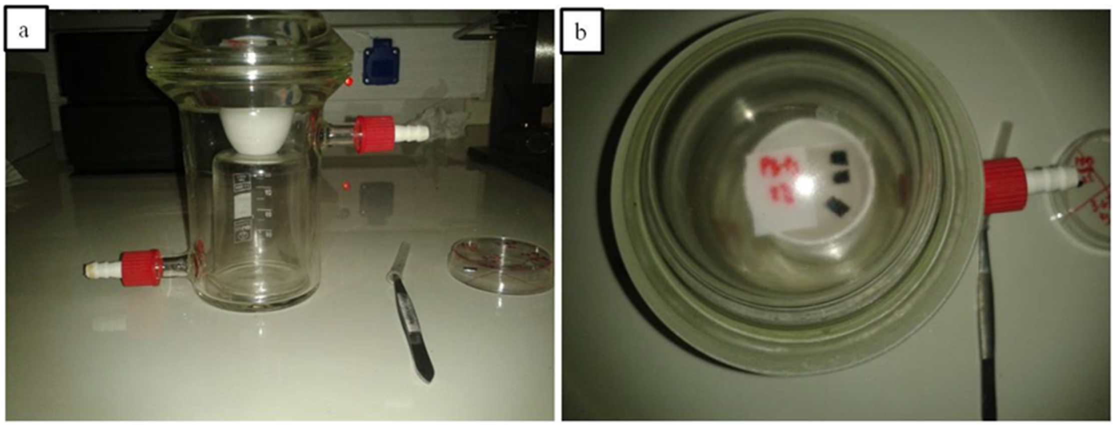

Figure 7. Side (**a**) and top (**b**) view of the improvised apparatus for the solvent vapor annealing procedure. Within this autoclave chamber, a glass substrate is evident up on which the Si wafers are deposited beneath which the solvent resides which stands on top of solvent deposit in a specific smaller chamber.

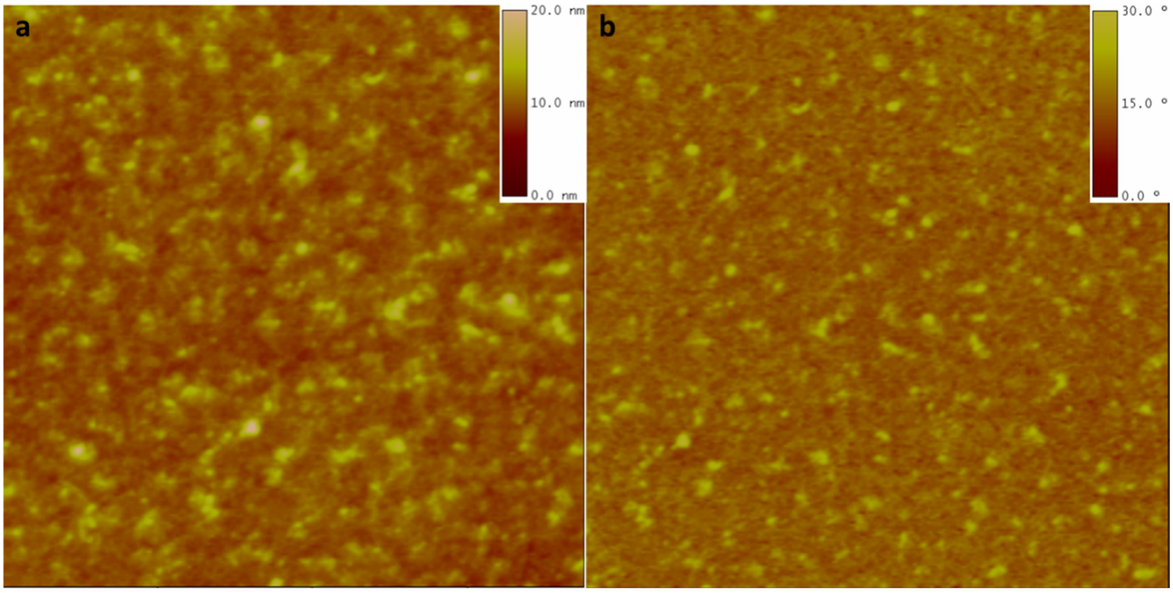

Figure 8. TP-AFM height (a) and phase (b) images for PS40-b-PB40_PS-γ-Fe_2O_3 composite thin film with loading rate 12.5% wt after 24h SVA.
